# Successful Removal of a Denture from the Œsophagus

**Published:** 1894-08

**Authors:** 


### Successful Removal of a Denture from the Œsophagus.

An extremely interesting account of the successful removal of a denture from the œsophagus is recorded in a recent number of the *Transactions of the Odonto-Chirurgical Society of Scotland.* The patient in question was a married woman, 28 years of age, and the teeth were swallowed during sleep, on the night of July 31, 1893. At the time of the accident she complained of considerable pain, and consulted a medical man, who ordered her removal to the Royal Infirmary, Edinburgh. On the following morning, when seen by Mr. David Wallace, she complained of slight pain opposite the manubrium sterni; she was able to swallow milk, but the act of deglutition gave rise to pain. With some difficulty the presence and situation of the foreign body was diagnosed, the exact position being gauged as "midway between the manubrium sterni and stomach." Œsophagotomy was performed, the opening into the gullet being made as low down as possible; dressing forceps were introduced and an attempt made, but without success, to grasp the plate. Laparotomy was then performed, the incision being in the median line between the xiphi-sternum and the umbilicus. Precautions were taken to prevent the passage of foreign matter into the general peritoneal cavity, and the stomach opened by an incision at right angles to the greater curvature, sufficiently long to admit the forefinger; but as some difficulty was encountered in finding the œsophageal opening, the incision was extended so as to admit the whole hand. The plate was found to be situated about one inch and a-half from the œsophageal opening, and was easily removed with suitable forceps and the wound dressed. *Journal of the British Dental Association.*

